# Effect of zinc-biofortified seeds on grain yield of wheat, rice, and common bean grown in six countries

**DOI:** 10.1002/jpln.201800577

**Published:** 2019-08-29

**Authors:** Abdul Rashid, Hari Ram, Chun-Qin Zou, Benjavan Rerkasem, Aildson P. Duarte, Simunji Simunji, Atilla Yazici, Shiwei Guo, Muhammad Rizwan, Rajinder S. Bal, Zhaohui Wang, Sudeep S. Malik, Nattinee Phattarakul, Rogerio Soares de Freitas, Obed Lungu, Vera L. N. P. Barros, Ismail Cakmak

**Affiliations:** 1 Pakistan Academy of Sciences, Islamabad, Pakistan; 2 Punjab Agricultural University, Ludhiana 141 004 Punjab, India; 3 Department of Plant Nutrition, China Agricultural University, Beijing 100193, P.R. China; 4 Plant Genetic Resource and Nutrition Lab., Chiang Mai University, Chiang Mai 50200, Thailand; 5 Agronomic Institute (IAC), Postal Box 28, 13012–970, Campinas, Brazil; 6 Golden Valley Agricultural Research Trust, P.O. Box 50834, Lusaka, Zambia; 7 Faculty of Engineering and Natural Sciences, Sabanci University, 34956 Istanbul, Turkey; 8 Jiangsu Key Laboratory for Solid Organic Waste Utilization, Nanjing Agricultural University, Nanjing, 210095, China; 9 Soil and Environmental Sciences, Nuclear Institute for Agriculture and Biology, Faisalabad, Pakistan; 10 Key Laboratory of Plant Nutrition and Agri-Environment in Northwest China, Collage of Natural Resources and Environment, Northwest A&F University, Yangling 712100, Shaanxi, China; 11 Department of Soil Science, University of Zambia, Lusaka, Zambia

**Keywords:** agronomic biofortification, *Oryza sativa*, *Phaseolus vulgaris*, soil zinc deficiency, *Triticum aestivum*, zinc-biofortified seeds

## Abstract

Seeds enriched with zinc (Zn) are ususally associated with better germination, more vigorous seedlings and higher yields. However, agronomic benefits of high-Zn seeds were not studied under diverse agro-climatic field conditions. This study investigated effects of low-Zn and high- Zn seeds (biofortified by foliar Zn fertilization of maternal plants under field conditions) of wheat (*Tritcum aestivum* L.), rice (*Oryza sativa* L.), and common bean (*Phaseolus vulgaris* L.) on seedling density, grain yield and grain Zn concentration in 31 field locations over two years in six countries. Experimental treatments were: (1) low-Zn seeds and no soil Zn fertilization (control treatment), (2) low-Zn seeds + soil Zn fertilization, and (3) Zn-biofortified seeds and no soil Zn fertilization. The wheat experiments were established in China, India, Pakistan, and Zambia, the rice experiments in China, India and Thailand, and the common bean experiment in Brazil. When compared to the control treatment, soil Zn fertilization increased wheat grain yield in all six locations in India, two locations in Pakistan and one location in China. Zinc-biofortified seeds also increased wheat grain yield in all four locations in Pakistan and four locations in India compared to the control treatment. Across all countries over 2 years, Zn-biofortified wheat seeds increased plant population by 26.8% and grain yield by 5.37%. In rice, soil Zn fertilization increased paddy yield in all four locations in India and one location in Thailand. Across all countries, paddy yield increase was 8.2% by soil Zn fertilization and 5.3% by Zn-biofortified seeds when compared to the control treatment. In common bean, soil Zn application as well as Znbiofortified seed increased grain yield in one location in Brazil. Effects of soil Zn fertilization and high-Zn seed on grain Zn density were generally low. This study, at 31 field locations in six countries over two years, revealed that the seeds biofortfied with Zn enhanced crop productivity at many locations with different soil and environmental conditions. As high-Zn grains are a by-product of Zn biofortification, use of Zn-enriched grains as seed in the next cropping season can contribute to enhance crop productivity in a cost-effective manner.

## Introduction

Zinc (Zn) deficiency is a predominant micronutrient disorder in crop plants and humans worldwide, adversely impacting crop productivity and human health (Bouis and Saltzman, 2017; Cakmak and Kutman, 2018). The cause of human Zn deficiency is ascribed mainly to inadequate dietary intake of Zn, primarily because of dependence on cereal-based diet low in concentration and bioavailability of Zn. Human Zn deficiency has been often observed in regions of the world where soils are usually deficient in Zn supplies (Cakmak, 2008; Cakmak et al., 2017). Wheat grain Zn concentration generally ranges from 20 to 35 mg kg^–1^ (Cakmak and Kutman, 2018), but in soils with very low Zn solubility due to low organic matter, calcareousness and high pH, grain Zn concentrations are even below 10 mg kg^–1^ (Graham et al., 1992; Erdal et al., 2002), *i.e.*, far below the target grain Zn concentration considered adequate for human nutrition (*i.e.*, 40–50 mg kg^–1^; Cakmak and Kutman, 2018).

Low grain Zn concentration has also adverse effects on its quality in terms of seed germination, seedling vigor and final yield (Rengel and Graham, 1995; Cakmak, 2008; Farooq et al., 2012). Seed reserves are the primary source of nutrients during early seedling development and should be at sufficient levels to support early growth, at least until the root system starts to mediate nutrient uptake from the soil (Welch, 1999). The effect of seed nutrient reserves on seedling development is more pronounced in nutrient-poor soils. Seed reserves of micronutrients are considered as ‘starter fertilizer’ and vital under soil conditions with their low supply, as well as under stress conditions such as drought stress (Rengel and Graham, 1995; Welch, 1999; Cakmak, 2008; Farooq et al., 2012). Impairments in crop growth and yield as a consequence of low-Zn seeds under field conditions are associated with poor seedling emergence and establishment as shown for wheat (Yilmaz et al., 1998; Cakmak et al., 1999; Harris et al., 2007). It is important to mention that low amounts of mineral nutrients in seeds may have irreversible adverse effects on seed vitality during seed development (Welch, 1999). Therefore, germination of such seeds may not be improved even with adequate application of the concerned nutrients in growth medium as shown for manganese (Mn) (Longnecker et al., 1996). These results indicate that seed coating or priming may not be always succsessful in improving seed germination and seedling development in case of the seeds which were harvested from mother plants with low vigor and vitality. It is obvious that enhancing seed Zn concentration under field conditions is of great importance both for addressing human Zn malnutrition and improving agronomic performance of seeds in Zn-deficient and marginal soils.

There are several options for enriching seeds with Zn. Seed treatment with Zn by priming or coating has been suggested as useful and cost-effective option for increasing seed Zn concentration (Harris et al., 2007; Farooq et al., 2012). Seed priming is the most commonly used strategy for realizing the effects of high-Zn seed on germination and seedling development, for example in maize (Harris et al., 2007; Imran et al., 2017), rice (Prom-u-thai et al., 2012), chickpea (Harris et al., 2008), wheat (Rehman et al., 2015), and barley (Ajouri et al., 2004). In recent field studies, priming of rice and wheat seeds with 0.5 M ZnSO_4_ improved number of productive tillers and grain yield (Rehman et al., 2018; Farooq et al., 2018). High-Zn seed is also helpful in minimizing adverse effects of environmental stresses on seedling growth such as drought and low temperature (Ajouri et al., 2004; Imran et al., 2013). Very recently, Candan et al. (2018) used Zn-enriched (biofortified) durum wheat seeds, by foliar sprays of Zn fertilizer to mother plants in field, and observed that Zn-biofortified seeds showed better germination and seedling development both under low soil Zn supply and drought stress conditions.

Besides the Zn enrichment of seeds by using priming, coating, or soaking approaches, seed Zn enrichment can be also achieved by foliar Zn fertilization of mother plants (Cakmak, 2008; Phattarakul et al., 2012; Zou et al., 2012; Jaksomsak et al., 2018). These findings indicate that better germination of seeds and seedling development might be under influence of sufficient amount of nutrients in seeds on the maternal plants. The present study was conducted to investigate the role of Zn-biofortified seeds, achieved under field conditions by foliar Zn sprays to mother plants, on seedling development and grain yield of wheat, rice, and common bean grown in 31 field locations over two years in six countries. To our knowledge, there is no published evidence about the role of high-Zn seed, achieved under field conditions by foliar Zn sprays to mother plants, on crop establishment and grain yield in field conditions under diverse soil types and agro-climatic conditions.

## 2 Material and methods

A series of field experiments on wheat, rice, and common bean were conducted over two cropping seasons at a total of 31 field sites in six countries. Wheat experiments were established in China, India, Pakistan, and Zambia, rice experiments in China, India and Thailand, and common bean experiments in Brazil. Tab. 1 summarizes some soil properties of the field locations and crop cultivars used in these experiments, and shows existence of large differences in soil properties among the locations as well as countries. Zinc concentrations in high-Zn and low-Zn seeds of all crops used in these experiments are given in Tab. 2. There were large differences in mean temperature and total rainfall during the corresponding cropping seasons which are presented in Tab. 3.

**Table 1:** Locations, years, initial soil properties including DTPA-extractable Zn and crop varieties used in the experiments with wheat, rice and common bean.

Crop/country	Year	Location (Field sites)	pH	DTPA Zn (mg kg^-1^)	NaHCO3 P (mg kg^-1^)	NH4OAc K (mg kg^-1^)	Organic carbon (%)	Variety
WHEAT								
China	2011–12	Hebei-Quzhou	7.8	0.33	11.7	111	0.13	Liangxing 99
		Shaanxi-Yongshou	7.8	0.37	14.0	166	0.14	Jimai 47
	2012–13	Hebei-Quzhou	8.2	0.40	15.3	92	0.15	Liangxing 99
		Shaanxi-Yongshou	7.8	0.37	12.8	140	0.12	Jimai 47
India	2011–12	Ludhiana	7.6	0.58	6.8	143	0.25	PBW 621
		Gurdaspur	7.5	0.55	7.1	148	0.29	PBW 621
		Bathinda	7.9	0.45	4.5	145	0.15	PBW 621
	2012–13	Ludhiana	7.6	0.58	6.8	143	0.25	PBW 621
		Gurdaspur	7.5	0.55	7.1	148	0.29	PBW 621
		Bathinda	7.9	0.45	4.5	145	0.15	PBW 621
Pakistan	2011–12	Faisalabad	8.3	0.56	5.1	120	0.29	Sehar-2006
		Muridke	8.0	0.45	5.4	84	0.30	Sehar-2006
		Kabirwala	8.1	0.52	9.8	110	0.38	Lasani-2008
	2012–13	Faisalabad	7.8	0.35	6.2	125	0.38	Faisalabad-2008
		Muridke	8.0	0.88	9.4	90	0.70	Faisalabad-2008
Zambia^a^	Both years	Chisamba	5.3	1.17	1.37	0.23	1.18	Lorrie II
	Both years	Chilanga	5.5	1.74	5.39	0.26	0.94	Lorrie II
RICE								
China	2011–12	Jiangsu-Rudong	8.2	1.38	12.97	97.7	0.19	Zhendao 11
	2012–13	Jiangsu-Rudong	8.4	0.82	12.97	97.7	0.19	Zhendao 11
India	Both years	Ludhiana	7.6	0.58	6.8	143	0.25	PR 120
	Both years	Gurdaspur	7.5	0.55	7.1	148	0.29	PR 120
Thailand	2013	Chiang Mai	6.2	0.90	54.4	58.5	0.87	Chainat 1
	2013	Takli	7.7	0.50	31.8	56.8	2.15	Chainat 1
COMMON BEAN^b^								
Brazil	2011–12	Capão Bonito	6.3	1.0	86	117.3	1.35	Perola
		Votuporanga	6.0	4.2	45	93.6	0.97	Perola
	2012–13	Capão Bonito	5.6	1.5	8	46.9	1.11	Perola
		Votuporanga	5.3	6.5	43	66.5	0.63	Perola

a For wheat in Zambia, soil NH_4_OAc K values are given in cmol kg–^1^.

b For common bean in Brazil, soil DTPA Zn values are given in mg dm–^3^; and P and K were extracted by anion exchange resin procedure (*van Raij* et al., 1986). The trials in Capão Bonito were conducted under rainfed conditions while the trials in Votuporanga were irrigated.

**Table 2:** Seed Zn concentration of wheat, rice and common bean used in the field experiments.

Crop/Country	Year	Location	Seed Zn concentration (mg kg^-1^)	
			Low-Zn seed	High-Zn seed
WHEAT				
China	2011–12	Hebei-Quzhou,	18	33
		Shaanxi-Yongshou	33	38
	2012–13	Hebei-Quzhou,	23	37
		Shaanxi-Yongshou	33	38
India	Both years	All (3) field locations	27	52
Pakistan	2011–12	All (3) field locations	30	50
	2012–13	Both field locations	19	41
Zambia	2011–12	Both field locations	23	46
	2012–13	Both field locations	15	43
RICE (un-husked paddy)				
China	2011	Jiangsu-Rudong	19	32
	2012	Jiangsu-Rudong	19	28
India	2012	Both locations	20	30
	2013	Both locations	21	35
Thailand	2013	Both locations	13	60
COMMON BEAN				
Brazil	2011–12	Both locations	38	47
	2012–13	Both locations	37	43

**Table 3:** Mean temperature and precipitation at field locations during crop growing periods in six countries.

Country	Year	Field location	Mean Temperature (°C)	Rainfall during crop growing season (mm)
WHEAT				
China	2011–12	Hebei-Quzhou	8.1	131
		Shaanxi-Yongshou	8.2	273
	2012–13	Hebei-Quzhou	8.1	172
		Shaanxi-Yongshou	9.0	143
India	2011–12	Ludhiana	18.7	124
		Bathinda	19.1	101
		Gurdaspur	17.8	93
	2012–13	Ludhiana	18.4	163
		Bathinda	18.7	63
		Gurdaspur	17.5	98
Pakistan	2011–12	Faisalabad	16.7	18
		Muridke	16.2	56
		Kabirwala	18.5	23
	2012–13	Faisalabad	17.8	99
		Muridke	15.5	90
Zambia	2011–12	Chisamba	17.7	0.0
		Chilanga	18.4	0.0
	2012–13	Chisamba	16.8	0.0
		Chilanga	17.2	0.0
RICE				
China	2012	Rudong-Jiangsu	23.1	122
	2013	Rudong-Jiangsu	23.9	110
India	2012	Ludhiana	29.6	382
		Gurdaspur	28.4	410
	2013	Ludhiana	29.3	732
		Gurdaspur	28.3	532
Thailand	2013	Chiang Mai	28.9	210
		Takli	27.9	197
COMMON BEAN				
Brazil	2011–12	Capão Bonito	19.9	685
	2012–13		19.9	477
	2011–12	Voruporanga	21.5	252
	2012–13		21.5	171

Seed rates used for wheat were as following: China, 188 kg ha^–1^ in 2011–12 and 202 kg in 2012–13; India, 100 kg ha^–1^; Pakistan, 125 kg ha^–1^; Zambia, 120 kg ha^–1^. In case of common bean, 13 seeds were sown m^–1^ row length in 45 cm apart rows. For rice, tranplanted seedling density was 307,500 hills ha^–1^ in China, 330,000 hills ha^–1^ in India, and 160,000 hills (3–4 sedlings per hill) ha^–1^ in Thailand. Field experiments at all locations comprised three Zn treatments: (1) local control treatment (*i.e.*, basal N, P, K fertilizers only, no Zn application), using low-Zn seeds, (2) local control treatment *plus* soil Zn application of 50 kg ZnSO_4_ × 7 H_2_O ha^–1^, using low-Zn seeds, and (3) local control treatment, using Zn-biofortified seeds. For these experiments, high-Zn seeds of all crops were obtained by two foliar sprays of ZnSO_4_ × 7 H_2_O to the previous year’s crops as described by Cakmak et al. (2010). In each experiment in a given country, low-Zn and high-Zn seeds of the same cultivar were used. Basal fertilizers (N, P, K) and soil Zn fertilizers were applied manually by broadcasting to the soil surface and incorporating into the surface soil (0–20 cm) with a tractor mounted cultivator, before planting.

All field experiments were laid out in randomized complete block design with four replications for wheat and rice and six replications for common bean. The doses and sources of applied N, P, and K were in accordance with the recommendation of local agriculture departments in the respective countries (Tab. 4). All crops received standard agronomic practices of the respective country, including locally recommended herbicides. Rice in China was intermittently irrigated. In India and Thailand, the fields were puddled, rice was transplanted and grown under flooded conditions. In all countries, wheat was irrigated. Because of low rainfall at Votuporanga in Brazil, common beanwas irrigated at this field location; at Capão Bonito location common bean was not irrigated. Wheat seedlings in all countries were counted within 1 m^2^ random area at 7–8 leaf stage, except for China where seedlings were counted 30 d after sowing. Common bean seedlings were counted over 1mrowlength in each plot 13 d after planting.

**Table 4:** Basal fertilizers applied and timing of N application in the field experiments.

Crop/Country	Basal nutrients (kg ha^-1^)			Nutrient sources			Timing of N fertilizer application^1^
	N	P	K	N	P	K	
WHEAT							
China	150	34.4	62.2	Urea	SSP^2^	K_2_SO_4_	1/2 N at sowing + 1/2 N at jointing stage
India	150	26.9	24.9	Urea	DAP^3^	KCl	1/2 N at sowing + 1/4 N at first irrigation 25 days after sowing (DAS) +1/4 at second irrigation (50 DAS)
Pakistan	120	34.4	0	Urea	DAP	-	1/2 N at sowing + 1/2 N at tillering
Zambia	168	25.8	24.9	Urea, DC^4^	DC	DC	30 kg N ha^-1^ at planting + 138 kg N ha^-1^ at tillering (28 DAS)
RICE							
China	200	34.4	62.2	Urea	DAP	K2SO4	1/2 N at transplanting + 1/2 N at tillering
India	150	17.2	0	Urea	DAP	KCl	1/3 N at transplanting + 1/3 N at 21 days after transplanting (DAT) + 1/3 N at 42 DAT
Thailand	150	34.4	0	Urea	TSP^5^	-	1/2 N at transplanting + 1/2 N at tillering (40-45 DAT)
COMMON BEAN							
Brazil							
Capão Bonito	83	34.4	50	Urea	TSP^6^	KCl	48 kg N ha^-1^ at planting + 35 kg N ha^-1^ 15–20 days after seedling emergence
Votuporanga	108	34.4	50	Urea	TSP^6^	KCl	48 kg N ha^-1^ at planting + 60 kg N ha^-1^ 15–20 days after seedling emergence

1 Unless stated otherwise, basal P and K were applied prior to crop sowing/transplanting.

2 SSP is single super phosphate;

3 DAP is di-ammonium phosphate;

4 DC is D compound;

5 TSP is triple superphoshate.

6 In Brazil, source of P was TSP, except for at Voruporanga location in 2013 where SSP *plus* TSP were applied to provide 44 kg S ha^–1^ because of low soil S level.

Grain yield was recorded at 13% moisture for wheat and at 14% moisture for rice and common bean. Grain samples were washed with tap water, then with deionized water, and dried at 45°C. The ground grain samples were digested by microwave-assisted wet digestion and Zn concentrations were analyzed using inductively coupled plasma optical emission spectrometry (ICP-OES; Vista-Pro Axial; Varian Pty Ltd, Mulgrave, Australia). Measurements of Zn were checked by using a certified standard reference material (SRM 1573a), obtained from the National Institute of Standards and Technology (Gaithersburg, MD, USA). Further details about preparation of grain samples for Zn analysis are given in Phattarakul et al. (2012) and Ram et al. (2016).

The data from each field location were analyzed using onefactor ANOVA and the means were separated by least significant difference (LSD) at P < 5%. For overall effectiveness, paired *t* test was performed to compare the datasets, locations, and years.

## 3 Results

### 3.1 Wheat seedling density

Across all wheat field locations in four countries over two years, minimum density of wheat seedlings was 105 m^–2^ and 101 m^–2^ with low-Zn seeds without and with soil Zn fertilization, respectively, at Chilanga location in Zambia (Tab. 5). Maximum seedling density was 491 m^–2^ at the Faisalabad location in Pakistan during 2012–13 with high-Zn seed without soil Zn fertilization. Even with local control treatment (*i.e.*, use of low-Zn seeds and no soil Zn fertilization), minimum and maximum density of wheat seedlings varied almost fourfold across 19 field locations in four countries during the 2011–12 wheat season, *i.e.*, from 105 seedlings m^–2^ to 436 seedlings m^–2^. The number of wheat seedlings per unit field area also varied as a consequence of Zn fertilization as well as of seed Zn concentration at some field locations (Tab. 5). For example, during 2012–13, soil Zn fertilization increased density of wheat seedlings at two field locations, one each in China and in Pakistan. Use of high-Zn wheat seeds resulted in denser plant population in most of the field sites in China and in all field sites in Pakistan (P < 5%). In wheat and common bean, seedling emergence was faster in the high-Zn seed plots.

**Table 5:** Wheat seedling density as affected by soil Zn fertilization and seed Zn concentration.^a^

Country	Year	Field location	Number of seedlings per m^-2^			LSD_0.05_
			Local control	Soil Zn fertilization	High-Zn seeds	
China	2011–12	Hebei-Quzhou	294 a	299 a	351 b	28
		Shaanxi-Yongshou	436	486	477	NS
	2012–13	Hebei-Quzhou	360 a	395 b	425 c	20
		Shaanxi-Yongshou	248 a	261 a	316b	24
		**Mean**	**334**	**360**	**392**	
India	2011–12	Ludhiana	211	210	214	NS
		Bathinda	200	198	213	NS
		Gurdaspur	201	202	215	NS
	2012–13	Ludhiana	199	192	209	NS
		Bathinda	193	200	198	NS
		Gurdaspur	198	198	195	NS
		**Mean**	**200**	**200**	**207**	
Pakistan	2011–12	Faisalabad	152 a	165 a	265 b	26
		Muridke	268 a	270 a	340 b	8
		Kabirwala	170 a	178 a	391 b	11
	2012–13	Faisalabad	244 a	235 a	491 b	32
		Muridke	139 a	162 b	245 c	14
		**Mean**	**195**	**202**	**346**	
Zambia	2011–12	Chisamba	169	180	168	NS
		Chilanga	105	101	125	NS
	2012–13	Chisamba	168	168	180	NS
		Chilanga	105	100	125	NS
		**Mean**	**137**	**140**	**146**	
		**Overall Mean**	**213**	**221**	**270**	
	Mean increase over control		–	3.57%	26.6%	
			P value of paired *t* test			
		Local control	–	0.706	0.331	
		Soil Zn fertilization		–	0.629	
		High-Zn seeds			–	

a Values of the same parameter within a row followed by different letters are significantly different (P < 5%).

### 3.2 Wheat and rice grain yield

Grain yield of wheat varied drastically across four countries and within various field locations of the same country and during two years (Tab. 6). For example, with low-Zn seeds and no soil Zn fertilization, maximum wheat grain yield of 8.38 t ha^–1^ was harvested at Hebei-Quzhou location in China and lowest grain yield of 2.55 t ha^–1^ was recorded at Muridke location in Pakistan. Soil application of ZnSO_4_ to the plots seeded with low-Zn seeds or using just high-Zn seeds led to grain yield increases at many field locations. Soil Zn application increased grain yield of wheat at one field site-year in China, all six site-years in India, and two site-years in Pakistan (Tab. 6). With soil Zn fertilization, a maximum grain yield increase of 28% was recorded at Kabirwala location in Pakistan during 2011–12. In Zambia, soil Zn fertilization did not increase wheat yield.

**Table 6:** Grain yield of wheat as affected by soil Zn fertilization and seed Zn density.^a^

Country	Year	Field location	Grain yield (Mg ha^-1^)			LSD_0.05_
			Local control	Zn fertilization	High-Zn seeds	
China	2011–12	Hebei-Quzhou	8.20 b	8.71 c	7.21 a	0.34
		Shaanxi-Yongshou	7.06	7.28	7.03	NS
	2012–13	Hebei-Quzhou	8.38	8.45	8.54	NS
		Shaanxi-Yongshou	2.65	2.57	2.18	NS
		**Mean**	**6.57**	**6.75**	**6.24**	
India	2011–12	Ludhiana	5.66 a	6.21 b	6.34 b	0.12
		Bathinda	4.74 a	5.32 b	5.47 b	0.21
		Gurdaspur	5.65 a	6.22 b	6.25 b	0.20
	2012–13	Ludhiana	5.62 a	5.99 b	5.74 ab	0.28
		Bathinda	4.57 a	4.90 b	4.84 b	0.14
		Gurdaspur	5.00 a	5.50 b	5.19 ab	0.41
		**Mean**	**5.30 a**	**5.77 b**	**5.71 b**	**0.30**
Pakistan	2011–12	Faisalabad	4.67	4.84	4.87	NS
		Muridke	4.34 a	4.47 a	5.40 b	0.65
		Kabirwala	4.14 a	5.30 c	4.74 b	0.46
	2012–13	Faisalabad	5.27 a	6.26 b	6.61 b	0.44
		Muridke	2.55 a	2.52 a	3.72 b	0.27
		**Mean**	**4.19 a**	**4.88 ab**	**5.07 b**	**0.75**
Zambia	2011–12	Chisamba	3.44	3.50	4.24	NS
		Chilanga	4.74	3.94	3.92	NS
	2012–13	Chisamba	4.54	4.12	4.75	NS
		Chilanga^b^	4.00	4.46	3.39	NS
		**Mean**	**4.18**	**4.00**	**4.08**	
		**Overall Mean**	**5.03**	**5.31**	**5.30**	
	Mean increase over control		–	**5.57%**	**5.37%**	
			P value of paired *t* test			
		Local control	–	0.752	0.674	
		Soil Zn fertilization		–	0.86	
		High-Zn seeds			–	

a Values of the same parameter within a row followed by different letters are significantly different (P < 5%).

b Data based on 3 replications.

In India, use of high-Zn wheat seeds improved grain yield at all field locations during 2011–12 and at one field location during 2012–13. In Pakistan, use of high-Zn seeds resulted in increased grain yield at all five wheat field sites over two years (Tab. 6), while use of high-Zn seeds did not increase grain yield in China and Zambia during both years. Highest increase in grain yield by using high-Zn seeds was found at Muridke location in Pakistan during 2012–13. Contrarily, use of high-Zn wheat seed caused a reduction in yield by 12% at Hebei-Quzhou location in China during 2011–12. Though mean increases in wheat grain yield with soil Zn fertilization of low-Zn seeds or using high-Zn seeds varied from country to country, overall mean increases with both treatments were very similar, *i.e.*, 5.57% with soil Zn fertilization and 5.37% with Zn-enriched seeds (Tab. 6).

Grain yield of paddy rice exhibited a large variation both among the countries studied and the field locations of a given country (Tab. 7). Soil Zn application increased paddy yield at both field sites in India during both years and at Takli field site in Thailand in 2013, but had minimal positive effect on paddy yield in China. Use of high-Zn rice seed resulted in increased paddy yield only at Rudong-Jiangsu location in China during 2013, where increase in paddy yield with Zn-rich seed was much greater (13.5%) than with Zn fertilization (5.5%) over control yield (Tab. 7). Despite 4.6-times higher Zn concentration with high-Zn rice seed used in Thailand (Tab. 2), this treatment did not improve rice productivity at both field locations in this country. It is important to mention that a seed Zn concentration of 60 mg Zn kg^–1^ for rice is unusual and it might be related to an unknown Zn contamination or experimental error.

**Table 7:** Paddy yield of rice (unhusked grains) as affected by soil Zn fertilization and seed Zn density.^a^

Country	Year	Field location	Paddy yield (Mg ha^-1^)			LSD_0.05_
			Local control	Soil Zn fertilization	High-Zn seed	
China	2012	Rudong-Jiangsu	7.77	7.80	8.50	NS
	2013	Rudong-Jiangsu	7.13 a	7.52 ab	8.09 b	0.84
		**Mean**	**7.45 a**	**7.66 ab**	**8.30 b**	**0.64**
India	2012	Ludhiana	4.62 a	5.03 b	4.70 ab	0.32
		Gurdaspur	4.97 a	5.46 b	5.30 ab	0.48
	2013	Ludhiana	5.30 a	5.88 b	5.48 ab	0.43
		Gurdaspur	6.11 a	6.45 b	6.17 a	0.23
		**Mean**	**5.26**	**5.70**	**5.40**	
Thailand	2013	Chiang Mai	6.67	6.84	7.03	NS
		Takli	4.22 a	4.51 bc	4.30 ac	0.26
		**Mean**	**5.45**	**5.68**	**5.67**	
		**Overall Mean**	**5.85**	**6.33**	**6.16**	
	Mean increase over control			**8.2%**	**5.3%**	
			P value of paired *t* test			
		Local control	–	0.500	0.803	
		Soil Zn fertilization	–	–	0.711	
		High-Zn seeds		–	–	

a Values of the same parameter within a row followed by different letters are significantly different (P < 5%).

### 3.3 Wheat and rice grain zinc concentration

In case of local control treatment (*i.e.*, low-Zn seeds and no soil Zn fertilization) there was a significant variability in seed Zn concentration in wheat both among the countries and among field sites of a given country (Tab. 8). The grain Zn concentration of the local control treatment varied from 13.5 mg kg^–1^ at Faisalabad in Pakistan to 36.1 mg kg^–1^ at Hebei-Quzhou in China over two cropping seasons. Wheat grain Zn concentration also showed a significant variation both with soil Zn application and with using high-Zn seeds among field locations. There were, however, very little increases in wheat grain Zn concentration with soil Zn fertilization as well as with using high-Zn seeds. Only at a few locations the increases in wheat grain Zn density were statistically significant such as at Hebei-Quzhou location in China during 2012–13, at Ludhiana location in India during 2011–12 and at all three field sites of Pakistan during 2011–12 (Tab. 8).

**Table 8:** Zinc concentration in wheat grains as affected by soil Zn fertilization and seed Zn density.^a^

Country	Year	Country and Location	Grain Zn concentration (mg kg^-1^)			LSD_0.05_
			Local Control	Soil Zn fertilization	High-Zn seed	
China	2011–12	Hebei-Quzhou	34.2	38.6	31.6	NS
		Shaanxi-Yongshou	21.7	22.3	19.4	NS
	2012–13	Hebei-Quzhou	36.1 b	39.6 c	32.1 a	2.7
		Shaanxi-Yongshou	19.6	22.4	18.6	NS
		**Mean**	**27.9**	**30.7**	**25.4**	**NS**
India	2011–12	Ludhiana	34.0 a	38.8 c	36.6 b	0.82
		Bathinda	28.1	28.6	29.2	NS
		Gurdaspur	31.9 a	32.3 a	35.3 b	1.8
	2012–13	Ludhiana	26.4	28.7	27.4	NS
		Bathinda	24.5	25.2	27.7	NS
		Gurdaspur	26.4 a	28.2 ab	28.9 b	2.4
		**Mean**	**28.4**	**30.2**	**30.4**	**NS**
Pakistan	2011–12	Faisalabad	13.5 a	28.9 c	19.4 b	5.1
		Muridke	19.9 a	25.2 b	23.8 b	3.6
		Kabirwala	21.5 a	28.0 bc	24.7 ac	5.8
	2012–13	Faisalabad	29.3	29.0	28.2	NS
		Muridke	32.9	31.0	28.4	NS
		**Mean**	**23.4 a**	**28.4ab**	**24.9 b**	**3.63**
Zambia	2011–12	Chisamba	26.8 a	34.0 b	29.0 ab	6.3
		Chilanga	23.2	23.8	22.5	NS
	2012–13	Chisamba	32.8	34.2	32.2	NS
		Chilanga	25.5 b	24.0 b	20.8 a	1.6
		**Mean**	**27.1**	**29.0**	**26.1**	
		**Overall Mean**	**26.4 a**	**29.6 b**	**27.1 a**	**1.9**
	Mean increase over control		–	**12.1%**	**2.65%**	
			P value of paired *t* test			
		Local control	–	0.097	0.881	
		Soil Zn fertilization		–	0.109	
		High-Zn seeds			–	

a Values of the same parameter within a row followed by different letters are significantly different (P < 5%).

Similar to wheat, Zn concentration of brown rice grains also varied among locations and years in the three countries, particularly in Thailand (Tab. 9). In Thailand, the same variety of rice (cv. Chainat 1) planted at two different locations during the same year produced grains with drastically different Zn concentrations. Despite producing lower yields at the Takli location, with all experimental treatments, compared to Chiang Mai location, rice grain Zn concentrations at Takli were much lower (7.4–10.2 mg kg^–1^) compared to Chiang Mai in Thailand (23.2–36.5 mg kg^–1^) as well as compared to all field locations in China and India (18.4–22.4 mg kg^–1^). Soil Zn fertilization of rice crop resulted in minimal increases in grain Zn concentration in China and India, while the increases were distinct and significant at both field locations of Thailand (Tab. 9). Use of high-Zn rice seeds caused only very slight increases in grain Zn density in all countries, although some of them are statistically significant (Tab. 9).

**Table 9:** Zinc concentration in brown rice grains as affected by soil Zn fertilization and seed Zn density.^a^

Country	Year	Field Location	Grain Zn concentration (mg kg^-1^)			LSD_0.05_
			Local Control	Soil Zn fertilization	High-Zn seeds	
China	2011	Rudong-Jiangsu	18.5 ab	19.1 b	17.4 a	1.2
	2012	Rudong-Jiangsu	18.7	19.4	20.2	NS
		**Mean**	**18.6**	**19.2**	**18.8**	
India	2012	Ludhiana	21.5	21.3	22.4	NS
		Gurdaspur	18.4 a	19.3 ab	20.0 b	1.8
	2013	Ludhiana	20.5	20.4	22.0	NS
		Gurdaspur	19.8	20.4	19.9	NS
		**Mean**	**20.2**	**20.3**	**21.4**	
Thailand	2013	Chiang Mai	23.2 a	33.9 b	36.5 b	4.1
		Takli	7.4 a	10.0 b	10.2 b	1.3
		**Mean**	**15.3**	**22.0**	**23.4**	
		**Overall Mean**	**18.5**	**20.5**	**21.1**	
Mean increase over control		–	–	**10.8%**	**14.0%**	
			P value of paired *t* test			
		Local control	–	0.344	0.599	
		Soil Zn fertilization		–	0.674	
		High-Zn seeds			–	

a Values of the same parameter within a row followed by different letters are significantly different (P < 5%).

### 3.4 Plant population, grain yield and grain zinc concentration of common bean

The density of common bean seedlings was not affected by Zn fertilization or seed Zn concentration. However, use of high-Zn seed resulted in increased grain yield at Capão Bonito location during 2012–13 (Tab. 10). Whereas mean grain yield of common bean (at two field locations over two years) was not affected by Zn fertilization, mean increase in grain yield by using high-Zn seed was 5% over local control (Tab. 10). Soil Zn fertilization improved grain Zn concentration of common bean at three field sites over two years. Also in case of common bean, using high-Zn seeds did not affect clearly grain Zn concentrations.

**Table 10:** Common bean seedlings density, grain yield and grain Zn concentration as affected by soil Zn fertilization and seed Zn density in four experiments conducted in Brazil.^a^

Year	Field location	Local control	Soil Zn fertilization	High-Zn seed	LSD_0.05_
**Number of seedling (m^-2^)**					
2011–12	Capão Bonito	9	9	12	NS
	Voruporanga	11	11	13	NS
2012–13	Capão Bonito	11	12	9	NS
	Voruporanga	9	9	9	NS
	**Mean**	**10**	**10**	**11**	
**Grain yield (Mg ha^-1^)**					
2011–12	Capão Bonito	1.53	1.54	1.52	NS
	Voruporanga	2.44	2.34	2.60	NS
2012–13	Capão Bonito	3.96 a*	4.15 ab	4.44 b	0.38
	Voruporanga	3.24	3.06	3.15	NS
	**Mean**	**2.79**	**2.77**	**2.93**	
Mean increase over control (%)		–	-0.7%	5.0%	
		P value of paired *t* test			
	Local control	–	0.977	0.869	
	Zn-fertilization		–	0.852	
	High-Zn seed			–	
**Grain Zn concentration (mg kg^-1^)**					
2011–12	Capão Bonito	29.1 a	31.3 b	30.8 b	1.6
	Voruporanga	35.6	38.6	36.9	NS
2012–13	Capão Bonito	25.5 a	27.8 b	24.5 a	1.2
	Voruporanga	34.5 a	37.0 b	34.1 a	1.4
	**Mean**	**31.2**	**33.7**	**31.6**	
Mean increase over control		–	8.0%	1.3%	
		P value of paired *t* test			
	Local control	–	0.492	0.586	
	Zn-fertilization		–	0.909	
	High-Zn seed			–	

a Values of the same parameter within a row followed by different letters are significantly different (P < 5%).

## 4 Discussion

This study investigated the effects of high-Zn seeds of wheat, rice, and common bean and soil Zn fertilization on crop yield and seed Zn concentration in commonly cultivated cultivars of these crops in six countries during two years at a total of 31 field locations having alkaline and acid soils (Tab. 1). Irrespective of experimental treatments, there was a large variation in grain yield of wheat and rice among countries and years, and even within field locations in a given country (Tabs. 6 and 7). The variation in grain yield might be ascribed, at least partially, to differences in soil and climatic conditions, yield potential of the cultivars used, and the crop management practices employed. It is known that the variations in soil chemical and physical properties have significant effects on grain yield of cereal crops (Yanai et al., 2001; Usowicz and Lipiec, 2017). It has been also documented that the subsoil constraints affect grain yield of wheat (McDonald et al., 2013).

The soils containing less than 0.5 mg kg^–1^ DTPA-extractable Zn are generally considered to be potentially Zn-deficient and could be responsive to soil Zn fertilization (Cakmak et al., 1996). There are also reports showing that the critical DTPA-extractable Zn for wheat and rice are below 0.9 mg kg^–1^ (Sims and Johnson, 1991; Prasad et al., 2013). Based on these criteria, most of the soils in wheat and rice field sites of the present study seemed to be potentially Zn-deficient (except for both field sites in Zambia in both years and one field site in China in 2011–12; Tab. 1). Therefore, there was an expectation for yield increases with soil Zn fertilization at most of the field locations. This was, however, true at lesser than expected field locations (Tabs. 6 and 7). Published evidence from around the world shows that grain yield of wheat and rice does not always correlate with DTPA-extractable soil Zn level, even below the 0.5 mg kg^–1^ level (Singh et al., 1987; Graham et al., 1999; Phattarakul et al., 2012; Zou et al., 2012; Duffner et al., 2014), most probably due to large influences of climatic and plant factors on grain yield in a given region with low Zn supply. For example, the magnitude of impairments in growth or yield under low soil Zn supply depends often upon soil moisture, seasonal climatic changes, and plant genotypes (Graham et al., 1992, Cakmak et al., 1997; Ekiz et al., 1998; Ma et al., 2017). At the Kabirwala location of Pakistan, soil Zn fertilization of wheat led to a substantial grain yield increase of 29% over the control treatment. There were also grain yield increases with soil Zn fertilization at all locations in India, some locations in China and at some other locations in Pakistan (Tab. 6), but the exceptionally high yield increase at Kabirwala location in Pakistan might be a consequence of higher responsiveness of wheat cv. Lasani-2008 to Zn fertilization (Tab. 1). In good agreement with these results, Nawaz et al. (2015), in a field study by using five commonly-cultivated wheat cultivars in Pakistan (*i.e.*, Bakhar-2002, Shafaq-2006, Sehar-2006, Faisalabad-2008, and Lasani-2008), showed that Lasani-2008 was the most responsive cultivar to Zn fertilization in terms of yield enhancement on a similar alkaline-calcareous soil having 0.36 mg kg^–1^ DTPA-extractable Zn.

Considering the critical Zn deficiency level in rice soils (Sims and Johnson, 1991; Prasad et al., 2013), the soils in all rice fields of this study (except for one field location in China in 2011–2012) were also potentially Zn-deficient (Tab. 1). At most rice field locations in three countries over two years, soil Zn fertilization or using Zn-biofortified seeds had positive effects on grain yield, and the effects were statistically significant at four field locations (Tab. 7). Thus, the effectiveness of DTPA soil testing for prognosis of Zn deficiency in rice crop was demonstrated in this extensive field research conducted in three countries over two years.

In case of common bean field trials in Brazil, soil Zn status appeared to be adequate, particularly at the Votuporanga location during both years (Tab. 1), and, overall, no yield increase was observed with soil Zn fertilization (Tab. 10). Though Zn fertilizer and high-Zn seed did not increase common bean plant population at both field locations during both years, grain yield was increased at a minimal level with Zn-biofortified seed at Capão Bonito location during 2012–13 (Tab. 10). Whereas no yield increase was observed with Zn fertilization, overall increase in common bean yield with high-Zn seed was 5.0% over control yield (Tab. 10). A similar observation was made by Hacisalihoglu et al. (2004) in a study with 35 common bean genotypes revealing that changes in seed Zn concentration did not show any relationship with the Zn deficiency tolerance of plants during early growth stage on a calcareous soil.

To our knowledge, this is the first study that has used the seeds of three staple food crops which were Zn-biofortified under field conditions by foliar Zn fertilization of mother plants in order to study the role of high-Zn seeds on plant polulation and grain yield under field conditions in six countries. At most of the field sites, using Zn-biofortified wheat seed had positive effect on number of seedlings per unit field area and resulted in denser plant population compared to the control treatment (*i.e.*, low-Zn wheat seed without Zn fertilization; Tab. 5). At seven locations over two years, the effect of high-Zn seeds on plant population density was statistically significant (Tab. 5). Denser crop stand establishments achieved by using Zn-biofortified seed led to significantly higher grain yields at field sites when compared to the local control treatment (Tab. 6). Thus, it was apparent that denser plant population and better tillering of wheat plants attained by using Zn-biofortified seeds contributed positively to better grain yield.

Despite the fact that the differences in Zn concentration in low-Zn seeds and high-Zn seeds used at all wheat field locations were usually substantial (Tab. 2), high Zn in seeds was not translated into denser plant stand establishment at some field locations (Tab. 5). For instance, despite almost two timee greater Zn concentration in high-Zn seed than in low-Zn seed in India (Tab. 2), wheat plant population did not increase significantly by using high-Zn seeds at any field location during both years, while grain yield was increased with the use of high-Zn seeds (Tab. 5). Additional research is required for better understanding of the minimal effect of Zn-rich wheat seed on number of seedlings per unit area in India.

Obviously, the effect of soil Zn fertilization on the number of wheat seedlings was much less than the effect obtained with using high-Zn seeds (Tab. 5). However, using high-Zn seeds and applying soil Zn fertilization were almost equally effective on grain yield of wheat when compared to the local control treatment (Tab. 6). It is obvious that the seeds biofortified with Zn under field conditions had a strong impact on seed vigor, seedling emergence, and crop establishment which in turn contributed to better wheat yield at most of the field locations. Previously, similar observations have been reported for manganese (Mn) in lupine plants, revealing that reduction in seedling emergence and development of abnormal seedlings, associated with low-Mn seeds, could not be eliminated by soil Mn fertilization (Crosbie et al., 1994; Longnecker et al., 1996). By contrast, seeds having already high Mn from maternal plants had better seed germination and did not show any impairment in seedling development. According to Welch (1999), low amount of micronutrients in seeds has detrimental effects on seed vigor and seedling development, even if the concerned micronutrients are applied to the growth medium during seed germination. It is suggested that if the seed micronutrients are low during the seed formation on maternal plants, vitality of those seeds is impaired at cellular or embryo level that cannot be corrected successfully by application of the concerned micronutrients during seed germination (Longnecker et al., 1996; Welch, 1999; Cakmak, 2008). In the present study, seed Mn concentrations of the experimental plants were sufficiently high, and there was no Mn deficiency problem in plants. The average seed concentration of the experimental plants was 42 mg kg^–1^ (ranging from 28 to 57 mg kg^–1^) for wheat, 24 mg kg^–1^ (ranging from 18 to 27 mg kg^–1^) for rice, and 30 mg kg^–1^ (ranging from 20 to 46) for common bean. These values are normal and adequate values for seed Mn when compared to the commonly found Mn concentrations in the corresponding crop plants (Marcar and Graham, 1986; Sperotto et al., 2013; Hacisalihoglu and Settles, 2013).

Although it was statistically significant (in case of wheat), soil Zn fertilization had minimal effect on grain Zn concentration in wheat, rice, and common bean (Tabs. 8, 9, and 10). Low effect of soil-applied Zn fertilizers on grain Zn has been already reported for several crops including wheat and rice (Phattarakul et al., 2012; Zou et al., 2012), confirming the results observed in this study. By contrast, foliar Zn application is required for attaining significant increases in grain Zn concentration to meet human needs. A foliar Zn treatment on high-Zn seeded crops was not included in the present study because the positive effects of foliar Zn sprays on grain Zn has been well documented in many earlier studies (Cakmak and Kutman, 2018).

High-Zn in seed has also protective effects against biotic and abiotic stress factors and improves crop stand establishment under stressful environments. Seedlings emerged from seeds containing low-Zn lose their ability to withstand environmental stresses, particularly in soils with low plant available Zn. Previously, Yilmaz et al. (1998) reported that the wheat seedlings emerged from high-Zn seeds in Zn-deficient soils under rainfed conditions were better in vigor, led to denser plant population, and resulted in higher grain yield. Very recently, it was reported that drought stress tolerance of young seedlings of durum wheat and *Nigella sativa* was improved by using seeds which were enriched with Zn either by foliar Zn fertilization of field-grown maternal plants (Candan et al., 2018) or through seed priming with Zn (Fallah et al., 2018), respectively. Priming of seeds with Zn was also found to be protective against salt stress during early seedling development (Imran et al., 2018). It appears that Zn provides additional benefits beyond its basic nutritional effects. Positive effects of Zn on the overall antioxidant capacity of plants are well-known (Cakmak, 2000). For example, increasing antioxidative potential of Zn-enriched seeds through the elevated activity of superoxide dismutase and catalase has been shown in wheat and *Nigella sativa* seedlings (Candan et al., 2018; Fallah et al., 2018). The positive effects of Zn priming of wheat and rice seed (in 0.5 mM ZnSO_4_ for 12 h) as well as seed Zn coating (with up to 1.5 g Zn kg^–1^ seed) were also reported on crop stand establishment and grain yield under field conditions (Ali et al., 2018; Farooq et al., 2018; Rehman et al., 2018). According to Harris et al. (2008), Zn priming of seeds is a highly useful and cost-effective farm practice and results in higher profit and better yield for growers as shown for chickpea and wheat under field conditions.

## 5 Conclusions

Today, increasing grain Zn concentration, especially in staple cereals, represents an important humanitarian challenge because dietary Zn deficiency, caused by low-Zn staple foods, is a growing concern in the developing world (Cakmak and Kutman, 2018), resulting in severe health complications and economic burden on healthcare system (Gödecke et al., 2018; Harding et al., 2018). The present study revealed that increasing concentration of Zn in seeds is also beneficial to seed germination, seedling vigor, crop stand establishment, and crop yield as observed under field conditions at many field locations of six countries. High-Zn seed is a by-product of biofortification strategy. Due to very critical agronomic and human health impacts, developing high-Zn seeds through plant breeding as well as agronomic approaches is highly desirable and required in addressing the issues of food and nutrition security.
